# Satb2 is required for the regionalization of retrosplenial cortex

**DOI:** 10.1038/s41418-019-0443-1

**Published:** 2019-10-30

**Authors:** Lei Zhang, Ning-Ning Song, Qiong Zhang, Wan-Ying Mei, Chun-Hui He, Pengcheng Ma, Ying Huang, Jia-Yin Chen, Bingyu Mao, Bing Lang, Yu-Qiang Ding

**Affiliations:** 1 Key Laboratory of Arrhythmias, Ministry of Education of China, East Hospital, and Department of Anatomy and Neurobiology, Collaborative Innovation Center for Brain Science, Tongji University School of Medicine, Shanghai, 200092 China; 2 State Key Laboratory of Medical Neurobiology and MOE Frontiers Center for Brain Science, Institutes of Brain Science, Fudan University, Shanghai, 200032 China; 3 State Key Laboratory of Genetic Resources and Evolution, Kunming Institute of Zoology, Chinese Academy of Sciences, Kunming, 650223 China; 4 Center for Excellence in Animal Evolution and Genetics, Chinese Academy of Sciences, Kunming, 650223 China; 5 Mental Health Institute of the Second Xiangya Hospital, National Clinical Research Center on Mental Disorders, National Technology Institute on Mental Disorders, Key Laboratory of Psychiatry and Mental Health of Hunan Province, Central South University, Changsha, 410011 Hunan China; 6 Department of Laboratory Animal Science, Fudan University, Shanghai, 200032 China

**Keywords:** Development, Neural ageing

## Abstract

The retrosplenial cortex (Rsp) is a transitional cortex located between the neocortex and archicortex, but the molecular mechanism specifying Rsp from the archicortex remains elusive. We here report that the transcription factor Satb2 is required for specifying Rsp identity during its morphogenesis. In Satb2 CKO mice, the boundary between the Rsp and archicortex [i.e., subiculum (SubC)] disappears as early as E17.5, and Rsp efferent projection is aberrant. Rsp-specific genes are lost, whereas SubC-specific genes are ectopically expressed in Rsp of Satb2 CKO mice. Furthermore, cell-autonomous role of Satb2 in maintaining Rsp neuron identity is revealed by inactivation of Satb2 in Rsp neurons. Finally, Satb2 represses the transcription of Nr4a2. The misexpression of Nr4a2 together with Ctip2 induces expression of SubC-specific genes in wild-type Rsp, and simultaneous knockdown of these two genes in Rsp Satb2-mutant cells prevents their fate transition to SubC identity. Thus, Satb2 serves as a determinant gene in the Rsp regionalization by repressing Nr4a2 and Ctip2 during cortical development.

## Introduction

In mammalian brain, the retrosplenial cortex (Rsp) is located in the caudomedial cerebrum between the neocortex and archicortex. The latter includes the hippocampal proper containing CA1–CA3, dentate gyrus (DG), and subiculum (SubC), which continues with the Rsp. In adult brain, the neocortex consists of six-layer cytoarchitecture, while the archicortex only comprises three layers. For Rsp, a transitional one between neocortex and archicortex, the lamination is simplified. Anatomically connected with the anterior thalamic nuclei, hippocampus, and visual cortex [1–3], the Rsp is considered as a key component of brain circuits regulating cognition including episodic memory, navigation, imagination [4–8], and the interaction between emotion and episodic memory [9].

Both Rsp and archicortex are generated in the medial pallium, and the primordium of archicortex is located ventral to that of Rsp. The cortical hem is situated more ventrally and secrets Wnts to specify hippocampal CA, DG, and SubC, but not adjacent Rsp [10, 11]. Early birth-dating studies have shown that Rsp neurons have a different birth sequence between medial and lateral parts from neocortex [12, 13], suggesting that the Rsp does not simply continue with neocortex. Because of the unique anatomical location, the Rsp is also an important object for studying the regionalization of archicortex from neocortex. Our and other previous studies have revealed that transcription factor Zbtb20 is critical for the specification of mouse hippocampal CA1 field identity, as shown by the fact that the Rsp and SubC shifts into hippocampal CA1 territory in Zbtb20 mutant mice [14, 15]. Conversely, transgenic expression of Zbtb20 in Rsp respecifies the Rsp neurons to CA1 neuron identity [16, 17]. However, the intrinsic molecular mechanism governing the regionalization of Rsp itself remains elusive.

The transcription factor Satb2 (special AT-rich DNA binding protein 2) is a major player in regulation of chromatin remodeling and gene expression via interacting with nuclear matrix attachment regions (MAR) [18, 19]. It is first identified as a disease gene for the cleft palate and other craniofacial abnormalities in human [20–23]. In addition, Satb2 is associated with multiple neurodevelopmental diseases including schizophrenia [24–28]. During embryonic development, Satb2 is essential for the establishment of the proper identity and axon projections of callosal neurons in mouse neocortex [29–31]. While postnatally, Satb2 is required for proper dendritic and soma adhesion of callosal neurons in neocortex [32]. In adult, Satb2 deletion leads to impairment in long-term memory [33, 34]. We also reported that mice with loss of Satb2 in cortex and hippocampus show hyperactivity, increased impulsivity, abnormal social novelty, and impaired spatial learning and memory [35]. In this study, we further explored the role of Satb2 in Rsp morphogenesis. Rsp neurons lost their identity and genes representing SubC identity expanded their expression into Rsp territory in Satb2 CKO mice. Satb2 maintains the Rsp identity by repressing the transcription of Nr4a2/Ctip2 cell autonomously, thus revealing a novel role of Satb2 in cortical development.

## Results

### Expression pattern of Satb2 in developing Rsp

**Fig. 1:**
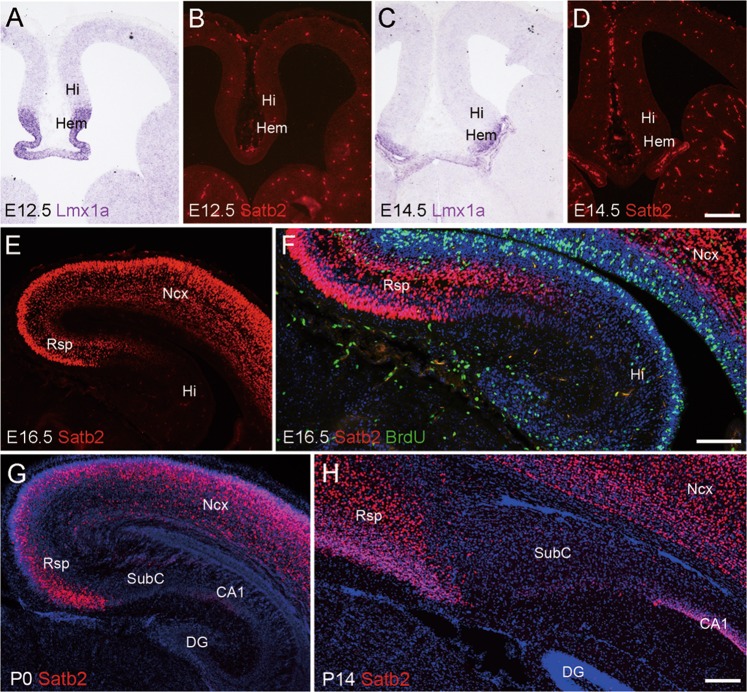
Expression of Satb2 in developing Rsp. **a**–**d** Satb2 is not expressed in the primordium of Rsp and hippocampus (Hi), or cortical hem (Hem) at E12.5 and E14.5. Cortical hem is shown by in situ hybridization for Lmx1a. **e** Satb2 is expressed in the presumptive Rsp but not hippocampal formation at E16.5. **f** Satb2^+^ cells are not labeled with BrdU in the Rsp at E16.5. **g** Expression of Satb2 is present in the Rsp and CA1 field but not SubC at P0. **h** Intense expression of Satb2 is observed in the Rsp, moderate in CA1 and weak in the ventral SubC at P14. DG dentate gyrus; Ncx neocortex. *n* = 3 mice for each stage. Scale bars = 200 μm in **a**–**e**, **g**, and **h** and 100 μm in **f**

We first examined the expression pattern of Satb2 in the Rsp using immunostaining during development. The expression of Satb2 was not observed in the primordia of the Rsp, hippocampus, or cortical hem (indicated by Lmx1a expression) at E12.5 and E14.5 (Fig. 1a–d). The expression of Satb2 emerged in the presumptive Rsp but not in the hippocampus at E16.5 (Fig. 1e). At P0, intense expression of Satb2 was observed in Rsp, and very weak expression was detected in hippocampal CA1, while no immunoreactivity was found in the SubC, CA2-CA3, or DG (Fig. 1g). At P4, P7, and P14, numerous Satb2^+^ cells with intense immunoreactivity were distributed throughout the Rsp and CA1 pyramidal layer, while some Satb2^+^ cells with weak immunoreactivity were observed in the ventral SubC without labeling in other subregions of the hippocampus (Fig. 1h and data not shown). In addition, no Satb2^+^ cells were labeled with BrdU injected 2 h before sacrifice of the pregnant mice at E16.5 (Fig. 1f). Thus, Satb2 is expressed in postmitotic Rsp neurons during embryonic development.

### Loss of Rsp identity in Satb2 CKO mice

**Fig. 2:**
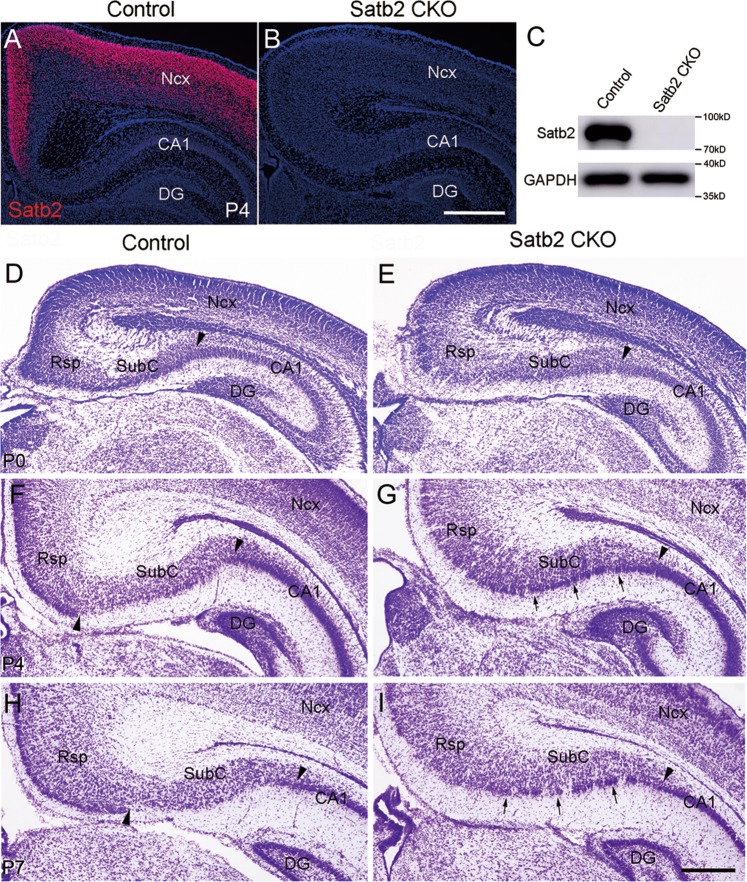
Morphological abnormalities in Rsp of Satb2 CKO mice. Satb2 immunoreactivity is observed in the cerebral cortex with high levels and hippocampal CA1 with low levels in control mice at P4 (**a**), but is absent in Satb2 CKO mice (**b**). **c** Western blots of Satb2 using cortical lysates from control and Satb2 CKO mice at P4. **d**, **e** No obvious differences in the Rsp, SubC, and hippocampus between control and Satb2 CKO mice at P0. The boundary between the Rsp and SubC (upward arrowheads) is clearly visible in P4 and P7 control mice (**f**, **h**) but absent in the CKO mice (**g**, **i**). Note that densely packed cells (arrows) are ectopically present in the ventral SubC, which joins the CA1 pyramidal cell layer in Satb2 CKO mice (**g**, **i**). Downward arrowheads indicate the boundary between the SubC and CA1 in both genotypes. DG dentate gyrus; Ncx neocortex. *n* = 3 mice for each stage of genotypes. Scale bar = 500 μm in **a** and **b** and 200 μm in **d**–**i**

Deletion of Satb2 in the cerebral cortex and hippocampus was confirmed by immunostaining and Western blotting in Emx1-Cre;Satb2^f/f^ (hereafter designated as Satb2 CKO) mice at P4 (Fig. 2a–c). Unlike conventional Satb2^−/−^ mice which die at birth, most Satb2 CKO mice survived to adult [35]. To explore possible roles of Satb2 in Rsp morphogenesis, we first examined the cellular architecture of Rsp in Satb2 CKO mice using Nissl staining. Our and other studies confirmed defective corpus callosum in Satb2 CKO mice [29–31, 35]. No obvious morphological differences in the Rsp, SubC, and hippocampus between control and Satb2 CKO mice at P0 (Fig. 2d, e). Notable morphological abnormalities were first observed at P4. At this stage, a clear boundary between the Rsp and SubC was observed in control mice, as shown by conspicuous superficial layers containing densely packed cells in the Rsp (upward triangle, Fig. 2f). In contrast, this boundary was absent in Satb2 CKO mice as shown by homogeneous distribution of cells in the Rsp region (Fig. 2g). This morphological abnormality was more evident in CKO mice at P7 than control (Fig. 2h, i). On the other hand, the boundary between the SubC and hippocampus was maintained (downward triangles, Fig. 2g, i), and the hippocampal components (CA1–CA3 and DG) were preserved in CKO mice (Fig. 2g, i and data not shown). Thus, the cellular architecture of the Rsp and SubC is changed in Satb2 CKO mice, particularly with a loss of the boundary between Rsp and SubC.

**Fig. 3:**
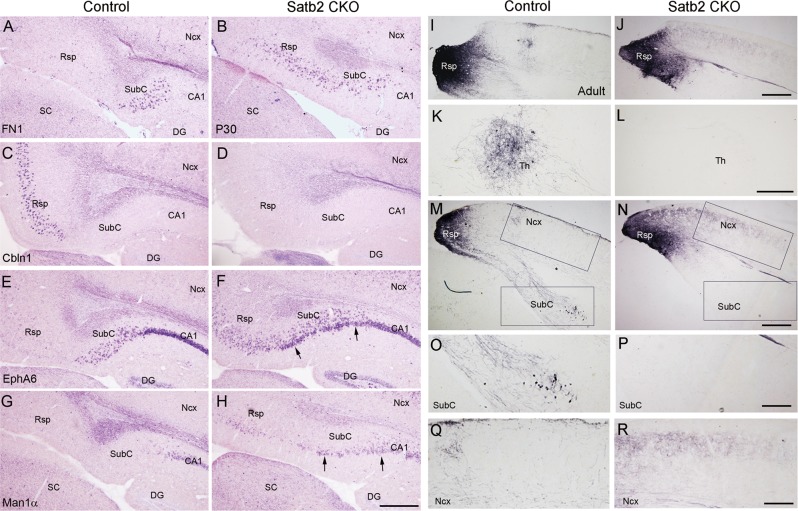
Rsp/SubC-specific gene expression is altered, and Rsp-associated efferent projection is aberrant in Satb2 CKO mice. At P30, FN1 expression is restricted to the SubC of control mice (**a**), but expands into the Rsp region of Satb2 CKO mice (**b**). Cbln1 is expressed in the Rsp of control mice (**c**) but absent in Satb2 CKO mice (**d**). **e**, **f** Intense EphA6 expression is present in CA1 pyramidal layer, and some EphA6^+^ cells with moderate signals are scattered in the SubC of control mice (**e**). In contrast, many EphA6^+^ cells with moderate signals are ectopically present in the Rsp region, and densely packed cells with intense signals (arrows) are ectopically located in the ventral SubC of Satb2 CKO mice (**f**). Man1α is selectively expressed in CA1 field of control mice (**g**), but many Man1α^+^ cells are present in the ventral SubC and the Rsp region of Satb2 CKO mice (arrows, **h**). **i**, **j** Injection sites of BDA in the Rsp of adult control and CKO mice. **k**–**r** Abundant BDA-labeled axon terminals are observed in ipsilateral anteroventral thalamic nuclei (**k**), and to less degree in ipsilateral SubC (**m**, **o**) in control mice. However, these projections are almost undetected in Satb2 CKO mice (**l**, **n**, **p**). Note that an increase of ipsilateral projection to neighboring cortex is observed in Satb2 CKO mice compared with control (**q**, **r**). DG dentate gyrus; Ncx neocortex; SC superior colliculus; Th thalamus. *n* = 3 mice for each genotype in ISH and *n* = 3 for each genotype in BDA tracing. Scale bars = 400 μm in **a**–**j**, **m**, and **n**, 200 μm in **k**, **l**, and **o**–**r**

We next performed in situ hybridization (ISH) using probes against a battery of genes specifically expressed in these regions in P30 mice with more matured brain. Expression of fibronectin 1 (FN1), a specific marker for SubC [14], was restricted to the SubC in control mice, but its domain expanded into the Rsp territory in Satb2 CKO mice (Fig. 3a, b). Notably, cerebellin 1 precursor protein (Cbln1) was only expressed in the control Rsp [36], whereas it was totally lost in Satb2 CKO mice (Fig. 3c, d). Many cells with intense EphA6 signals were located in the CA1, some with moderate signals in the SubC, and few with faint signals, if any, in the Rsp of control mice (Fig. 3e). In Satb2 CKO mice, however, numerous EphA6^+^ cells with moderate signals were ectopically present in the Rsp, and some densely packed cells with intense signals formed a band in the ventral subicular region (arrows, Fig. 3f). Furthermore, mannosidase 1 alpha (Man1α), a marker for mature CA1 pyramidal neurons [37], was expressed in the CA1 but not the Rsp or the SubC of control mice (Fig. 3g). In contrast, although Man1α expression in the CA1 field was not obviously changed, many Man1α^+^ cells were ectopically present in the Rsp and subicular region of Satb2 CKO mice (Fig. 3h). In addition, the Rsp has strong projections to anterior thalamic nuclei and hippocampus [5]. We stereotactically injected biotinylated dextran amine (BDA) into the Rsp of adult mice (Fig. 3i, j) and observed numerous BDA-labeled axons in the anteroventral thalamic nuclei (Fig. 3k) and to less degree in the SubC ipsilaterally in control mice (Fig. 3m, o, data not shown), but few were observed in these regions of Satb2 CKO mice (Fig. 3l, n, p). Besides, an increase of ipsilateral projection to adjacent neocortex was observed in Satb2 CKO mice (Fig. 3m, n, q, r). Taken together, these results indicate that Satb2 is required for Rsp neurons to establish their identity.

### Abnormal gene expression in Rsp of Satb2 CKO mice

To explore possible mechanism underlying the misspecification of Rsp during development, we focused on gene expression at P4, the earliest stage showing obvious morphological abnormality by Nissl staining in Satb2 CKO mice (Fig. 2f, g).

**Fig. 4:**
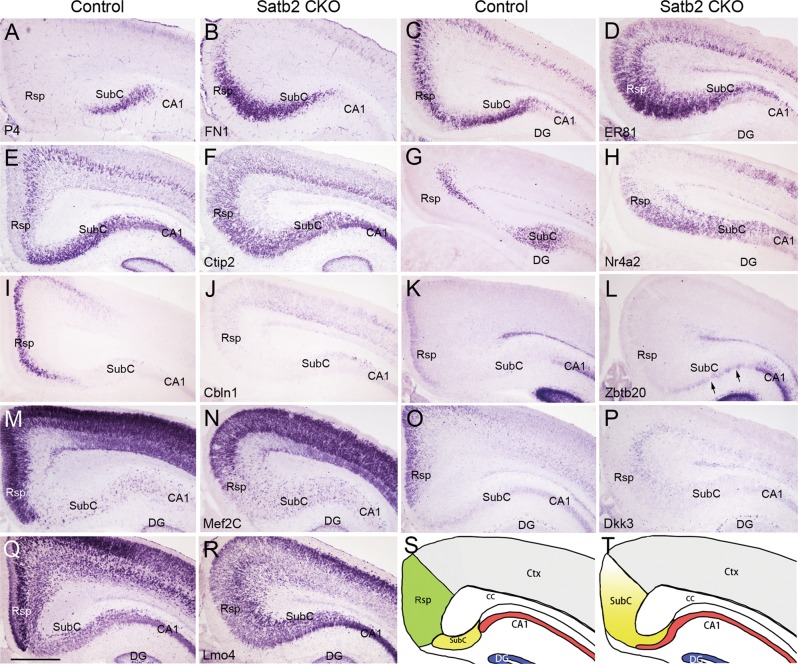
Rsp neurons lose their identity in Satb2 CKO mice. FN1 is restricted to the SubC of controls (**a**) but expressed by neurons in the Rsp region of Satb2 CKO mice at P4 (**b**). **c**–**h** ER81, Citp2, and Nr4a2 are expressed in the SubC and specific layers of the Rsp in control mice (**c**, **e**, **g**). In Satb2 CKO mice, however, they are expressed throughout the Rsp regions while remain largely unchanged in the SubC (**d**, **f**, **h**). Cbln1 shows restricted expression in the Rsp of control mice (**i**), but its expression is lost in Satb2 CKO mice (**j**). Zbtb20 is expressed in superficial Rsp and CA1 of control mice (**k**), but undetectable in the Rsp region while remains unchanged in the CA1 of Satb2 CKO mice (**l**). Note that ectopic Zbtb20^+^ cells (arrows) are present in the subicular region of Satb2 CKO mice. Mef2C is intensely expressed in the Rsp and weakly in the SubC of control mice (**m**), but its expression was largely reduced in the Rsp region to a comparable cellular density of the SubC in Satb2 CKO mice (**n**). Dkk3 is strongly expressed in upper Rsp of control mice (**o**) but absent in the Rsp region of Satb2 CKO mice (**p**). Intense Lmo4 expression is reduced in the Rsp regions with a similar intensity to that in the SubC in Satb2 CKO mice (**r**) compared with controls (**q**). **s**, **t** Diagram of Rsp fate change in Satb2 CKO mice. DG dentate gyrus. *n* = 3 mice for each genotype. Scale bar = 500 μm

Like P30 brain, FN1 expression was restricted to the SubC of P4 control mice, and its ectopic expression in the Rsp region was evident in Satb2 CKO mice (Fig. 4a, b). ER81 was expressed in the SubC and only in the middle portion of control Rsp (Fig. 4c), but it was expressed throughout the Rsp of mutant mice (Fig. 4d). Similarly, Ctip2 was located in the SubC and only in deep Rsp in control mice, while it was observed throughout the Rsp with no obviously altered expression in the SubC of Satb2 CKO mice (Fig. 4e, f). Finally, Nr4a2 was expressed in the SubC and the deepest Rsp in control mice, but its expression expanded to the entire Rsp in CKO mice (Fig. 4g, h).

Next, we moved to examine genes that specifically or highly expressed in the Rsp. Cbln1 was selectively expressed in the Rsp of control mice, but its expression was totally lost in the Rsp region of Satb2 CKO mice (Fig. 4i, j). Zbtb20 was weakly expressed in the superficial Rsp and the CA1 of control mice (Fig. 4k). However, its expression was lost in the Rsp region, and some densely stained cells were ectopically located in the ventral SubC in Satb2 CKO mice (arrows, Fig. 4l). Mef2C was intensely expressed in the Rsp but weakly in the SubC of control mice (Fig. 4m). In CKO mice, however, Mef2C expression in the Rsp became weak but comparable to that in the SubC (Fig. 4n). Consistent with previous reports [38, 39], Dkk3 was intensely expressed in the upper portion of control Rsp, but its expression disappeared in mutant mice (Fig. 4o, p). Expression of Lmo4 was present in both the Rsp and SubC with the highest intensity in the middle portion of Rsp in control mice, but the highest expression no longer existed while the expression in the SubC remained unchanged in Satb2 CKO mice (Fig. 4q, r). Further, we checked gene expression earlier than P4. Expression patterns of FN1, Ctip2, Nr4a2, Dkk3, Sox5, and Mef2C showed similar changes at P0 (Fig. S1) and E17.5 (Fig. S2) in the Rsp and SubC of Satb2 CKO mice compared with those at P4.

Taken together, Rsp-specific genes are undetectable, SubC-specific genes expand into the Rsp, and those expressed in both lose their unique pattern in the Rsp and change to subicular expression pattern in Satb2 CKO mice from E17.5, supporting the idea that Rsp neurons lose their identity in the absence of Satb2. The diagram of fate change of Rsp, SubC, and CA1 in Satb2 CKO mice is shown in Fig. 4s, t.

### Satb2 maintains Rsp identity cell autonomously

**Fig. 5:**
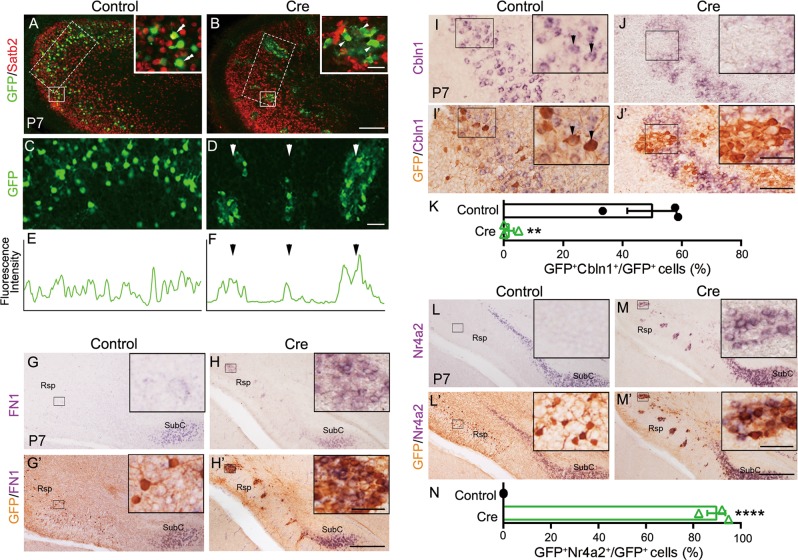
Deletion of Satb2 in a subset of Rsp neurons leads to ectopic expression of SubC-specific genes. Rsp neurons of Satb2^f/f^ mice were transfected with GFP alone or GFP plus Cre-expression plasmid by in utero electroporation at E14.5 and analyzed at P7. **a** GFP-expressing neurons (green) are evenly distributed in the Rsp and adjacent neocortex, and some of them are immunostained with Satb2 antibody (double arrowheads, insert) in Satb2^f/f^ mice. **b** Cre-expressing (Satb2-mutant) neurons (green) are not immunostained with Satb2 antibody (arrowheads, insert). **c**–**f** Knockout of Satb2 in Rsp neurons results in clumping of cell bodies, instead of even distribution. **g**, **h**′ Clumping Satb2-mutant neurons initiate expression of the SubC-specific gene FN1 in the Rsp (**h**′), but control neurons do not (**g**′). For more clear signals, mRNA signals were photographed first, and then immunostaining of GFP was performed and imaged again. **i**–**j**′ Clumping Satb2-mutant neurons located in the middle portion of Rsp fail to express the Rsp-specific gene Cbln1 (**j′**) but control neurons do so (arrowheads, **i**′). **k** Quantitation of Cbln1-expressing GFP^+^ cells out of total GFP^+^ cells. About half of GFP^+^ control cells express Cbln1, while very few GFP^+^ mutant cells do so. **p < 0.01 (*n* = 3), error bars represent S.E.M. **l**–**m**′ GFP-transfected (control) cells do not have ectopic Nr4a2 expression in the Rsp of Satb2^f/f^ mice (**l**′), but Cre-expressing Satb2-mutant cells do so **m**′. **n** Quantitation of Nr4a2-expressing GFP^+^ cells out of total GFP^+^ cells. ****p < 0.0001 (*n* = 3), error bars represent S.E.M. Scale bars = 50 μm in inserts of **b**, **h**′, **j**′, and **m**′, 200 μm in **b**, 50 μm in **d**, and 400 μm in **h**′, **j**′, and **m**′

Satb2 is essential for the establishment of callosal neuron identity [29–31]. The ectopic expression of deep-layer genes (e.g., Ctip2, ER81, and Sox5) in the superficial layer, and loss of expression or downregulation of superficial-layer-specific/enriched genes (e.g., Mef2C) suggest that there are similar regulations of these genes by Satb2 between the neocortex and Rsp, raising the possibility that Satb2 is not only required for maintaining Rsp identity but also for preventing Rsp neurons to adopt SubC identity. To explore this, we performed the deletion of Satb2 in a fraction of Rsp neurons via in utero electroporation (IUE) of Cre- and EGFP-expressing plasmids into Satb2^f/f^ embryos at E14.5 and examined pups at P7. EGFP was used to show Cre-expressing cells [32, 40]. Deletion of Satb2 by Cre misexpression in Satb2^f/f^ mice was confirmed by immunostaining for Satb2 (Fig. 5b). Electroporation of control plasmid in Satb2^f/f^ mice resulted in homogeneous distribution of EGFP-labeled neurons throughout the Rsp (Fig. 5a, c, e). In contrast, Cre-transfected neurons clumped in the Rsp of Satb2^f/f^ mice (Fig. 5b, d, f), consistent with our previous finding by in vivo knocking down Satb2 in the neocortex [32].

As in Fig. 4a, FN1 is restricted to the SubC of EGFP-delivered Satb2^f/f^ mice without its transcripts in the Rsp (Fig. 5g, g′). Remarkably, Cre/EGFP-expressing neurons initiated FN1 expression in the Rsp (Fig. 5h, h′). We next examined whether expression of Cbln1, the Rsp-specific gene, was changed in the Rsp. Cbln1 is in the middle portion of Rsp, and about half of control GFP^+^ neurons expressed Cbln1 (Fig. 5i, i′, k). Conversely, GFP^+^ clumping Satb2-mutant neurons in the middle portion of Rsp failed to express Cbln1, but adjacent neurons without Cre kept Cbln1 expression (Fig. 5j, j′, k). Thus, it is likely that Satb2 is not only required for maintaining Rsp neuronal identity but also for preventing them to take SubC identity.

The unique role of Satb2 in the Rsp was also explored by examining Nr4a2, which is abundantly expressed in the SubC and is sparsely expressed in the deepest Rsp at P4 (Fig. 4g). Cre/EGFP expression in Rsp neurons resulted in initiation of Nr4a2 expression in these cells regardless of their location in the Rsp of Satb2^f/f^ mice (Fig. 5m, m′, n), and this ectopic expression was not observed in EGFP-transfected cells (Fig. 5l, l′, n). Taken together, our results indicate a cell-autonomous role of Satb2 in maintaining Rsp identity.

### Satb2 is required for establishment of Rsp identity by repressing Nr4a2/Ctip2

**Fig. 6:**
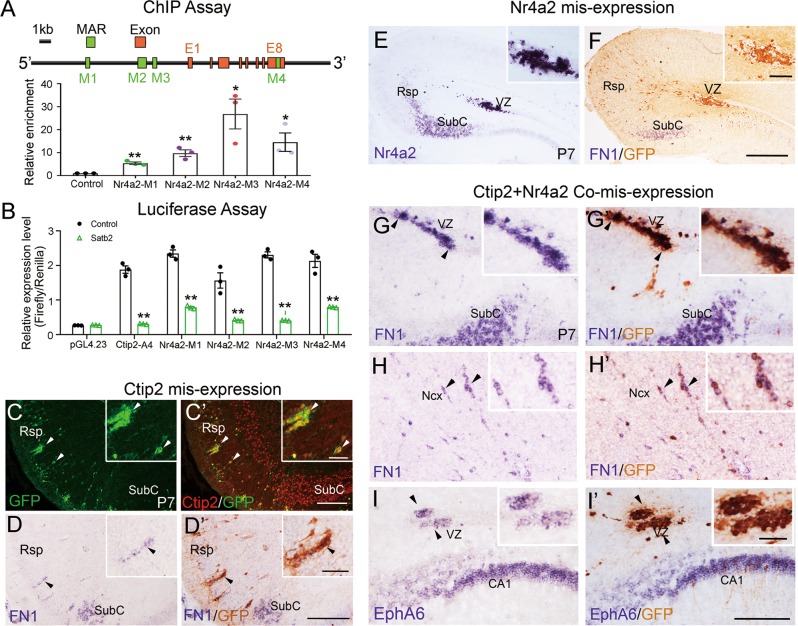
Satb2 determines Rsp fate by repressing Ctip2 and Nr4a2. **a** Diagram of Satb2-binding MARs in Nr4a2 genomic locus. In vivo ChIP assay using embryonic mouse cortex shows a remarkable enrichment of Satb2-binding MARs in Nr4a2 locus. *p < 0.05, **p < 0.01 (*n* = 3), error bars represent S.E.M. **b** Luciferase reporter assay shows that the transcriptional activities of Ctip2-A4 (positive control) and four Nr4a2 MARs are significantly reduced in the presence of Satb2. **p < 0.01 (*n* = 3), error bars represent S.E.M. **c–i**′ Ctip2- and/or Nr4a2-expression plasmids together with GFP are delivered into Rsp neurons by in utero electroporation in wild-type mice at E14.5 and pups are analyzed at P7. To make signals more clearly to compare, mRNA signals were photographed first, and then immunostaining of GFP was performed and photographed again. **c**, **c**′ Ctip2 is misexpressed in wild-type Rsp, and some of them are clumped. Inserts show Ctip2 expression in GFP^+^ cells (arrowheads). **d**, **d**′ A few Ctip2-misexpressed neurons contain FN1 transcripts and they often reside in ventral Rsp (arrowheads). Most of Rsp neurons with misexpression of Nr4a2 are arrested in VZ (**e**), and none of them contains FN1 transcripts in wild-type Rsp (**f**). Rsp neurons with co-misexpression of Ctip2 and Nr4a2 initiate FN1 expression in the VZ of Rsp (**g**, **g**′) and neocortical region (**h**, **h**′). **i**, **i**′ EphA6 is also ectopically induced in wild-type Rsp neurons when Ctip2 and Nr4a2 are co-misexpressed. VZ ventricular zone; Ncx neocortex. *n* = 3 mice for each group. Scale bars = 200 μm in **c**′, **d**′, and **i**′, 50 μm in inserts of **c**′, **d**′, and **i**′, 400 μm in **f**, and 100 μm in inserts of **f**

In Satb2 CKO or Cre-transfected Satb2^f/f^ mice, two genes Nr4a2 and Ctip2 were strongly upregulated in Rsp Satb2-mutant cells (Figs. 4 and 5). Previous studies showed that Satb2 directly binds to MAR of Ctip2 genome and suppresses its expression [29, 30]. To investigate whether Satb2 did so to Nr4a2, we performed ChIP and luciferase assays. By SMARTest prediction (Genomatix), we identified four putative MARs in Nr4a2 genome (Fig. 6a). Using ChIP assay and qPCR, we found marked enrichment of these Satb2-binding MARs (Fig. 6a). To test if Satb2 regulates the transcription of Nr4a2, the dual luciferase assay was performed to examine the transcription activity of Satb2 by binding different MARs in Nr4a2 genome. Ctip2-A4 was previously found as a Satb2-binding MAR [29] and used as a positive control. Nr4a2 MARs showed significantly reduced luciferase transcription in the presence of Satb2 (Fig. 6b), suggesting a repressive role of Satb2 in regulating Nr4a2 expression, similar to the effect of Satb2 on Ctip2. This is further supported by the data that none of Satb2^+^ neurons were positive for Nr4a2 and only a small proportion of Satb2^+^ neurons expressed Ctip2 in the Rsp of wild-type mice at P7 (Fig. S3).

Next, we moved to examine whether misexpression of Nr4a2 and/or Ctip2 in wild-type Rsp mimics the phenotypes found in Satb2 CKO mice. First, Ctip2 plus GFP plasmids were misexpressed in the Rsp of wild-type mice by IUE at E14.5 and pups were examined at P7. Ctip2 was expressed in almost all GFP-positive cells (arrowheads, Fig. 6c′). Importantly, SubC-specific gene FN1 was induced in Ctip2-misexpressing cells, although it was only present in a small fraction (less than 5%) (arrowheads, Fig. 6d, d′). GFP^+^/FN1^+^ cells were only observed in the ventral Rsp proximal to the SubC. These results suggest that upregulation of Ctip2 alone cannot fully account for the loss of Rsp identity in Satb2 CKO mice. Similarly, misexpression of Nr4a2 alone failed to induce FN1 expression in Rsp, and most of Nr4a2-misexpressing neurons were arrested in the VZ of Rsp (Fig. 6e, f). Finally, Nr4a2/Ctip2 expression plasmids were co-electroporated into Rsp, and coexpression of Ctip2 and Nr4a2 was confirmed by immunostaining (Fig. S4). The majority of Nr4a2/Ctip2-misexpressing cells expressed FN1 in the Rsp, although a large number of neurons were arrested in the VZ (Fig. 6g′, h′). Cell counts showed that about 90% of Nr4a2/Ctip2-misexpressing cells in the VZ and about 70% of them in the cortex contained FN1 transcripts. In addition, EphA6 was also ectopically induced in Nr4a2/Ctip2-misexpressing cells of the Rsp (Fig. 6i, i′). Thus, simultaneous misexpression of Nr4a2/Ctip2 can force Rsp neurons to initiate expression of SubC-specific genes, so the loss of Rsp identity in Satb2 CKO mice can be partially explained by upregulated expression of Nr4a2/Ctip2 in the Rsp.

**Fig. 7:**
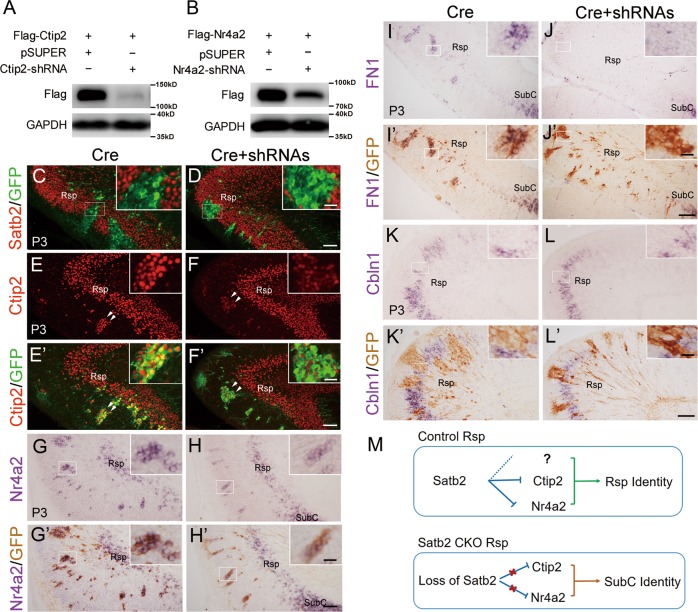
Knocking down Ctip2 and Nr4a2 in Rsp Satb2-mutant cells prevents their fate transition. **a**, **b** The efficiency of shRNAs for Ctip2 and Nr4a2. Ctip2 (**a**) and Nr4a2 (**b**) protein are greatly reduced when cotransfected with respective shRNA in HEK293T cells. **c–l**′ GFP + Cre or GFP + Cre + shRNAs were electroporated into the Rsp of Satb2^f/f^ mice at E14.5 and mice were examined at P3. Satb2 immunofluorescence is absent in GFP^+^ cells transfected with Cre (**c**) and Cre + shRNAs (**d**). **e**–**f**′ Ctip2 immunofluorescence in Cre + shRNAs-cotransfected cells is greatly reduced (**f**, **f**′) compared with those transfected with Cre only (**e**, **e**′). **g**–**h**′ In situ hybridization signals for Nr4a2 in Cre + shRNAs-cotransfected cells are greatly reduced (**h**, **h**′) compared with those transfected with Cre only (**g**, **g**′). To make signals more clearly to compare, mRNA signals were photographed first, and then immunostaining of GFP was performed and imaged again. **i**–**j**′ In situ hybridization of FN1 shows that FN1 is ectopically expressed in Cre-transfected cells (**i**, **i**′) but absent in Cre + shRNAs-cotransfected cells (**j**, **j**′). **k**–**l**′ No in situ hybridization signals for Cbln1 are observed in Cre- or Cre + shRNAs-cotransfected cells. *n* = 3 mice for each group. Scale bars = 25 μm in inserts of **d**, **f**′, **h**′, **j**′, and **l**′, and 200 μm in **d**, **f**′, **h**′, **j**′, and **l**′. **m** Proposed model showing the role of Satb2 in determination of Rsp identity during morphogenesis

We next examined whether the fate change could be prevented by knocking down Nr4a2 and/or Ctip2 in Satb2-mutant cells. Constructs of short hairpin RNA (shRNA) against Nr4a2 and Ctip2 were generated, and the efficiency of shRNAs was verified by the data that Flag-tagged Nr4a2 and Ctip2 were dramatically reduced when cotransfected with respective shRNA (Fig. 7a, b). We then tested Ctip2-shRNA or Nr4a2-shRNA with Cre/EGFP expression plasmids in the Rsp of Satb2^f/f^ mice at E14.5 and checked pups at P3. Still a very small fraction of Cre + EGFP + Ctip2-shRNA- or Cre + EGFP + Nr4a2-shRNA-cotransfected cells with ectopic expression of FN1 was observed (Fig. S5c-d′). Then co-electroporation of both shRNAs for Nr4a2 and Ctip2 (shRNAs) with Cre/EGFP was performed. Satb2 was deleted in GFP^+^ cells in the Rsp of both Cre- and Cre + shRNAs-electroporated Satb2^f/f^ mice (Fig. 7c, d), and the expression of Nr4a2 and Ctip2 was greatly reduced in Cre + shRNAs-cotransfected cells compared with those transfected with Cre only (Fig. 7e–h′). As mentioned above, SubC-specific FN1 was ectopically expressed in Cre-transfected cells in the Rsp (Fig. 7i, i′), whereas no detectable FN1 was found when Cre + shRNAs was co-electroporated (Fig. 7j, j′), indicating the transition of cell fate from Rsp to SubC was blocked. Finally, Rsp-specific marker Cbln1 was examined and no Cbln1 was detected in Cre + shRNAs-cotransfected cells and this is also the case for Cre-transfected cells (Fig. 7k–l′). In summary, knockdown of Nr4a2/Ctip2 in Rsp Satb2-mutant cells prevents their fate transition to SubC although they could not fully regain Rsp fate, possibly due to the residual expression of Nr4a2 and/or Ctip2, or other unidentified genes regulated by Satb2 (Fig. 7m).

## Discussion

In this study, we examined the transitional cortex Rsp of Satb2 CKO mice and revealed for the first time that Satb2 is required for the regionalization of Rsp during development.

The cerebral cortex is anatomically and functionally divided into different areas, such as the motor, somatosensory, and visual cortices. During cortical morphogenesis, the regionalization of cortex is a critical step in cortical development and great progresses have been made, such as FGF8 signaling and gradient expression of transcription factors in this process [41–44]. In this study, we found that Rsp neurons lose their identity as evidenced by the loss of laminated architecture, Rsp efferent projections, and Rsp-specific gene expression. The expression of Satb2 is initiated in postmitotic Rsp neurons around E16.5, and the abnormal gene expression reflecting the failure of Rsp neuron specification was observed at E17.5, showing that Satb2 is a postmitotic determinant gene for Rsp morphogenesis. Since Satb2 is not expressed in subicular neurons until P4 and inactivation of Satb2-induced SubC-specific FN1 expression is only present in the Rsp but not adjacent neocortex, the role of Satb2 in maintaining Rsp identity cannot be simply explained by consequences of global cortical changes caused by Satb2 deficiency, suggesting different developmental mechanisms between Rsp and adjacent neocortex. In consistent with this, birth-timing between neurons from medial and lateral parts is distinct between Rsp and neocortex [12, 13].

The role of Satb2 in neocortical development has been established. Callosal neurons in the superficial layers require Satb2 to establish their identity, as these neurons fail to project to the contralateral cortex but start to express deep-layer-specific genes in Satb2-mutant mice [29–31]. Given that Rsp takes subicular fate in Satb2 CKO mice, we speculate that Satb2 in Rsp neurons may also suppress genes that specify the subicular fate, but so far, no reports are available concerning genes that determine the SubC identity. We found that Ctip2 is ectopically expressed by superficial Rsp neurons in Satb2 CKO mice, and Ctip2-misexpression in wild-type Rsp initiates the expression of FN1 in a few Rsp neurons. Thus, the upregulation of Ctip2 alone cannot fully account for the phenotype observed in Satb2 CKO mice. On the other hand, Nr4a2 is likely to be another target gene, as the expansion of Nr4a2-expression domain from the SubC into the Rsp territory occurs as early as E17.5. Although misexpression of Nr4a2 alone does not induce FN1 expression, co-misexpression of Ctip2 and Nr4a2 can induce FN1 and EphA6 transcription in wild-type Rsp neurons. Our in vitro data also indicate that Satb2 suppresses Nr4a2/Ctip2 transcription by binding to their MAR. Furthermore, knockdown of Nr4a2 and Ctip2 in Satb2-mutant cells prevents the transition of cell fate from Rsp to SubC although these cells did not fully regain the Rsp cell fate. Based on these results, we propose that Satb2 prevents the SubC from expansion into the Rsp territory by suppressing the expression of Ctip2/Nr4a2 in the morphogenesis of Rsp.

Considering the critical roles of Satb2 in the morphogenesis of neocortex [29–32] and transitional cortex (this study) as well as important roles of the hippocampus in learning and memory, further studies are needed to explore if Satb2 plays a role in the differentiation of CA1 neurons and establishment of neuronal networks within the hippocampal formation and with other brain regions. Recently, we found that emotional behavior and spatial learning and memory were impaired in Satb2 CKO mice [35]. Rsp- or CA1-specific Cre is needed to exclusively delete Satb2 for elucidating its function in these regions, which will promote our understanding of how Satb2 is involved in neurodevelopmental diseases and psychiatric disorders [33].

## Materials and methods

### Animals

Satb2 targeted ES cells (EPD0098_3_H05) purchased from the International Mouse Phenotyping Consortium were used to generate Satb2 knockout-first mice, which were initially crossed with Flper mice to obtain floxed Satb2 mice. To conditionally knock out Satb2 gene exclusively in the cerebral cortex and hippocampus, Emx1-Cre mice [45] were then crossed with floxed Satb2 mice to delete exon 4 (Emx1-Cre;Satb2^f/f^; Satb2 CKO). In the offspring, these genotypes (e.g., Satb2^f/+^ or Satb2^f/f^) were used as controls. Both sexes were used for experiments. We chose *n* = 3 animals for each different experiment experientially because the phenotypes were observed in all CKO mice (over 20 with different ages) we examined. Animal care practices and all experiments were reviewed and approved by the Animal Committee of Tongji University School of Medicine, Shanghai, China.

### DNA constructs

Ctip2 shRNA was targeted against 5′-CCATAGACTCTCCTGCCAT−3′ (nucleotides 1335–1353 of NM_001079883); Nr4a2-shRNA was targeted against 5′-GGACCTCACCAACACTGAA−3′ (nucleotides 577–595 of NM_013613). These shRNA plasmids were constructed as described previously [32]. In brief, the complementary oligonucleotides were annealed and inserted into the pSUPER-EGFP vector (pSUPER for short), which itself alone is used as a control for Ctip2-shRNA and/or Nr4a2-shRNA.

Specific primers containing Flag coding sequence were used to amplify Ctip2 CDS (NM_001079883) or Nr4a2 CDS (NM_013613) from mouse cortical cDNA library. The PCR products were cloned into the pCAGGS vector to obtain pCAG-Flag-Ctip2 and pCAG-Flag-Nr4a2 constructs.

### Immunohistochemistry, BrdU labeling, ISH, and Nissl staining

Embryos at different stages and mice at different postnatal ages were perfused with 4% paraformaldehyde and brains were dissected out. After cryoprotection with 30% sucrose, brains were sectioned into 25-μm-thick slices using a cryostat (CM1950, Leica, Wetzlar, Germany). For immunohistochemistry, the following primary antibodies were used: rabbit anti-Satb2 (1:300; ab92446, Abcam, Cambridge, UK), rat anti-Ctip2 (1:300; ab18465, Abcam), goat anti-Nr4a2 (1:200; AF2156, R&D system, Minneapolis, MN, USA), and goat anti-GFP (1:2000; NB100–1770, Novus Biologicals, Centennial, CO, USA). Species-specific Alexa Fluor 488- or Cy3-conjugated antibodies were used to detect primary antibodies. Briefly, brain slices were incubated with primary antibodies at 4 °C overnight and with secondary antibodies at room temperature for 3 h, followed by incubation with streptavidin-Cy3 (1:1000; 016160084, Jackson ImmunoResearch, West Grove, PA, USA) at room temperature for 1 h. Slices were counterstained with Hoechst 33258 (1:2000; 94403, Sigma, St. Louis, MO, USA). For BrdU labeling, pregnant mice received one pulse of BrdU (100 mg/kg body weight; B9285, Sigma) and were euthanized 2 h later. Brain slices were sequentially subjected to treatment in sodium citrate (0.01 M, pH6.0) at 95 °C for 10 min, HCl (2 N) at 37 °C for 20 min, and sodium borate (0.1 M, pH 8.5) at room temperature for 10 min. Slices were then immunostained with mouse anti-BrdU antibody (1:300; NA61, Calbiochem, Burlington, MA, USA) as described above. For all immunostaining experiments, three animals for each group/genotype were used.

Antisense digoxigenin-labeled RNA probes were synthesized, and ISH was performed as described previously [46]. The following RNA probes were used: EphA6, FN1, Man1α, Mef2C, Zbtb20 [14], Ctip2, ER81, Lmo4, Cbln1, Sox5, Dkk3, and Nr4a2 (Allen Brain Atlas) and Lmx1a (nucleotides 316–795 of NM_033652). Nissl staining was performed as described previously [47]. For all ISH and Nissl staining experiments, three animals for each group/genotype were used.

For double labeling of ISH and GFP immunostaining, slices were subjected to ISH procedures first. After visualization for mRNA, slices were incubated with goat anti-GFP antibody at 4 °C overnight, followed by biotinylated horse anti-goat IgG for 3 h. Slices were then processed using ABC kit (1:500; PK4000, Vector Laboratories, Burlingame, CA, USA) for 1 h and immunoreactivity was visualized by incubation with diaminobenzidine and H_2_O_2_. Images were captured using an epifluorescence microscope (Eclipse 80i, Nikon, Tokyo, Japan). For all double ISH and immunostaining experiments, three animals for each group/genotype were used.

### BDA tracing

Under anesthesia with sodium pentobarbital, mice were injected with BDA (1%; Invitrogen, Grand Island, NY, USA) into the Rsp of adult mice at age of 8 weeks. One week later, mice were euthanized and perfused with 4% paraformaldehyde. Brain slices were incubated with ABC kit (1:500; Vector Laboratories) and BDA labeling was visualized with DAB-based reaction with nickel intensification as described previously [48]. For BDA tracing experiments, three animals for each group/genotype were used.

### HEK293T cell cultures and Western blots

Cell cultures and Western blots were performed as described previously [32]. Briefly, HEK293T cells were cultured in Dulbecco’s modified Eagle’s medium (Gibco, Grand Island, NY, USA) containing 10% fetal bovine serum (Hyclone, Pittsburgh, PA, USA). Cells of about 80% confluence were transfected using Lipofectamine 2000 (Invitrogen) according to the manufacturer’s instructions. The ratio of shRNA plasmid to expression vector is 2:1. Forty-eight hours later, cells were lysed in ice-cold RIPA buffer containing protease inhibitor cocktail (Thermo Scientific, Grand Island, NY, USA). Mouse cortical tissue from P4 control and Satb2 CKO mice was lysed in the same lysis buffer. The lysates were then loaded on SDS-PAGE, transferred, probed with rabbit anti-Flag antibody (1:3000; Cell Signaling Technology, Danvers, MA, USA), rabbit anti-Satb2 antibody (1:1000; ab92446, Abcam), or mouse anti-GAPDH antibody (1:2000; Santa Cruz, Dallas, TX, USA), developed with species-specific horseradish peroxidase-conjugated secondary antibodies (1:3000; KangChen, Shanghai, China), and visualized with enhanced chemiluminescence (KisLab, Shanghai, China). For all Western blots, three cultures/animals for each group/genotype were used.

### In utero electroporation

For delivery of Cre-expressing plasmids, male and female Satb2^f/f^ mice were crossed. Timed pregnant Satb2^f/f^ mice (E14.5) were deeply anesthetized and embryos were surgically manipulated as described previously [32, 49]. pCAG-Cre and control empty plasmids (1 μg/μl) were injected directly into the lateral ventricles of the Satb2^f/f^ embryos’ brains. To label electroporated cells, pCAG-EGFP plasmid (1 μg/μl) was co-injected with pCAG-Cre or control plasmids. For misexpressing Ctip2 or Nr4a2 in Rsp region, pCAG-Flag-Ctip2, pCAG-Flag-Nr4a2, or control empty plasmid (2 μg/μl) with pCAG-EGFP plasmids (1 μg/μl) were injected directly into the lateral ventricles of the wild-type embryos at E14.5. For coexpression of Ctip2 and Nr4a2, pCAG-Flag-Ctip2 and pCAG-Flag-Nr4a2 (2 μg/μl each) with pCAG-EGFP plasmid (1 μg/μl) were introduced. To knock down Ctip2 and/or Nr4a2 in Satb2-mutant Rsp cells, pCAG-Cre (1 μg/μl) and pCAG-EGFP plasmids (1 μg/μl) together with Ctip2-shRNA (2 μg/μl), Nr4a2-shRNA (2 μg/μl) or both shRNAs were injected directly into the lateral ventricles of Satb2^f/f^ embryos.

Five square electric pulses (36 V) with 50-ms duration were then delivered through the uterus at 1-s interval using forcep-type electrodes, connected with an electroporator (ECM830, BTX, Holliston, MA, USA). Pups were euthanized at P3 or P7 for analysis. For IUE experiments, three animals for each group/genotype were used.

### ChIP and luciferase reporter assays

ChIP assay was performed using E16.5 WT mouse cortices with Satb2 antibody (ab34735, Abcam). Coprecipitated DNA were purified with SimpleChIP Plus Sonication Chromatin IP Kit (Cell Signaling Technology), and relative DNA abundance of regions of interest was measured by qPCR. Primer sequences are listed as follows: Nr4a2-MAR1-F: TGTCAAACACTTGTTGCATGAGT, Nr4a2-MAR1-R: AGTCCCACAAATTAGCACTGGT; Nr4a2-MAR2-F: CCCAGCCTGTTTTTCACCAAG, Nr4a2-MAR2-R: AGTGGCATGAGGCTTTACCAT; Nr4a2-MAR3-F: CATGATACTAGCACATACTAAGCCA, Nr4a2-MAR3-R: CGATGGGATTACACTTATGTTTGCT; and Nr4a2-MAR4-F: AGAATCACATGCTTGTCCCCT, Nr4a2-MAR4-R: CTAAATGTTGCGTGGGTGGC. For ChIP experiments, three animals were used.

For luciferase reporter assay, pGL4 dual transcription activity luciferase system (Promega, Madison, WI, USA) was used. Medulloblastoma cell line DAOY was cultured in 24-well plates and transfected by Lipofectamine 2000 (Invitrogen). DAOY for each well was transfected with 400 ng Ctip2-MAR4-luc or Nr4a2-MAR(1–4)-luc reporter constructs, 50 ng pGL4.73 construct (as internal control), together with 400 ng pCAG-Satb2 or its empty constructs. The cells were harvested for the luciferase reporter assays 30 h after transfection. For luciferase experiments, three cultures for each group were used. HEK293T and DAOY cell lines were kindly provided by Stem Cell Bank, Chinese Academy of Sciences, and are routinely tested for mycoplasma contamination.

### Statistical analysis

For quantitation of Satb2^+^ neuronal subtypes in Rsp, Rsp area (about −2.70 mm to −2.92 mm) was divided into three equal parts in the horizontal direction, and the middle part was selected as the interested region. The number of Satb2^+^, Ctip2^+^, Nr4a2^+^, and double-labeled cells was counted, and the ratios were calculated. For quantification of electroporated cell number in Rsp, the retrosplenial granular cortex was selected as the interested region. The number of GFP^+^, Cbln1-expressing GFP^+^ cells, and Nr4a2-expressing GFP^+^ cells was counted, and respective ratios were calculated. For comparison of the distribution of electroporated cells in Rsp, the color-profiler plugin for ImageJ was used to generate plots of fluorescence intensity.

All experiments were replicated at least three times. Origin8 software was used for statistical analysis. Comparisons were performed using two-tails Student’s *t* tests or Wilcoxon signed rank test. *p* values of less than 0.05 were considered statistically significant. Error bars represent s.e.m.

## Supplementary information

Supplemental figure legends

Figure S1

Figure S2

Figure S3

Figure S4

Figure S5
