# Supplementary material for: Satb2 is required for the regionalization of retrosplenial cortex
Source: Cell Death Differ. 2019 Oct 30;27(5):1604–17. doi: 10.1038/s41418-019-0443-1 (PMC7206047; doi:10.1038/s41418-019-0443-1)
Supplement: Supplementary file 1 — Supplemental figure legends [file 41418_2019_443_MOESM1_ESM.docx]

**Supplementary figure legends**

**Fig. S1** Altered gene expression in Rsp of Satb2 CKO mice at P0. (A, B) FN1 is restricted to the SubC of controls (A) but ectopically expressed in the Rsp region of Satb2 CKO mice (B). (C, D) Citp2 is not expressed in superficial Rsp in control mice but is observed throughout the Rsp region in Satb2 CKO mice. Note that Citp2 expression in the SubC is unchanged in Satb2 CKO relative to controls. (E, F) Dkk3 expression is intense in superficial and moderate in deep Rsp of control mice (E), but it is only observed in the deepest Rsp region of Satb2 CKO mice (F). (G, H) Mef2C expression in the Rsp regions is dramatically reduced in Satb2 CKO mice (H) compared with controls (G). n=3 mice for each genotype. Scale bar = 300 μm.

**Fig. S2** Rsp neurons lose molecular identity in Satb2 CKO mice at E17.5. (A, B) Nr4a2 expression is restricted to the SubC of control mice (A), but ectopically located in the Rsp region of Satb2 CKO mice (B). (C, D) Expression of Dkk3 in the Rsp region is undetectable in Satb2 CKO mice (D) compared with controls (C). Noted that Dkk3 expression in the pia matter is unchanged. (E, F) Expression of Mef2C in the Rsp region is dramatically reduced in Satb2 CKO mice (F) relative to controls (E). (G-J) Ctip2 (G, H) and Sox5 (I, J) expression is primarily distributed in deep Rsp of control mice (G, I) whereas it is observed throughout the Rsp of Satb2 CKO mice (H, J). (K, L) Similar expression of Zbtb20 in presumptive hippocampus is observed in both control and Satb2 CKO mice. Note that Zbtb20 is not detected in the Rsp of the two genotypes. Inserts are high magnifications of the Rsp regions of corresponding panels. n=3 mice each genotype. Scale bars = 50 μm in inserts of A-L and 200 μm in A-L.

**Fig. S3** The expression pattern of Satb2-/Ctip2- or Nr4a2-expressing cells in Rsp at P7. (A) Co-immunostaining of Satb2 and Ctip2 in Rsp. (B) The statistical ratios of Satb2- and Ctip2-expressing cells in Rsp. (C) Co-immunostaining of Satb2 and Nr4a2 in Rsp. (D) The statistical ratios of Satb2- and Nr4a2-expressing cells in Rsp. n=3 mice for each double immunostaining. Scale bars = 50 μm in inserts of A, C and 200 μm in A, C.

**Fig. S4** Mis-expression of Ctip2 and Nr4a2 by *in utero* electroporation at P7 in wild-type mice. (A-C) Double immunostaining of Ctip2 and Nr4a2 in VZ and cortex of electroporated mice. VZ, ventricular zone, Ncx, neocortex. n=3. Scale bars = 100 μm.

**Fig. S5** A few cells with FN1 transcripts are found in Cre+Ctip2-shRNA- or Cre+Nr4a2-shRNA-transfected Rsp of Satb2^f/f^ mice. (A, B) Loss of Satb2 in GFP/Cre/Ctip2-shRNA^+^ (A) or GFP/Cre/Nr4a2-shRNA^+^ (B) cells in Satb^2f/f^ mice. (C, C’) A few Cre+Ctip2-shRNA-neurons (arrowhead) contain FN1 transcripts. (D, D’) A few Cre+Nr4a2-shRNA-neurons (arrowhead) contain FN1 transcripts. n=3 for each group. Scale bars = 50 μm in inserts of A-F and 200 μm in A-F.
