# Supplementary figures and images for: Satb2 is required for the regionalization of retrosplenial cortex
Source: Cell Death Differ. 2019 Oct 30;27(5):1604–17. doi: 10.1038/s41418-019-0443-1 (PMC7206047; doi:10.1038/s41418-019-0443-1)

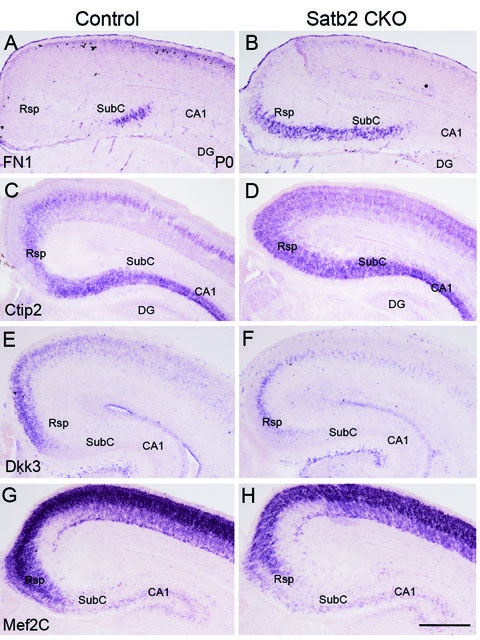

Supplement: Supplementary file 2 — Figure S1 [file 41418_2019_443_MOESM2_ESM.jpg]

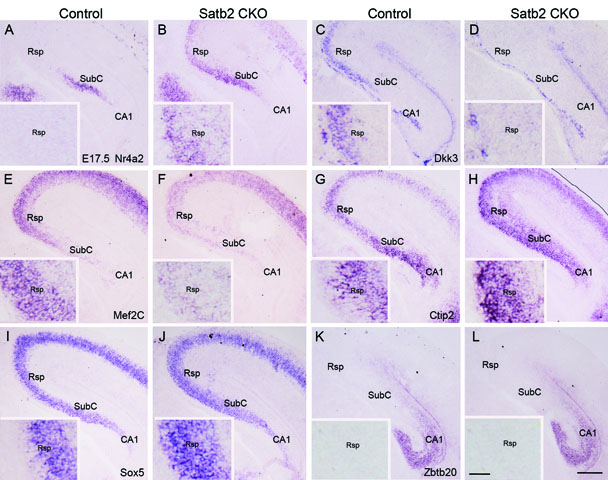

Supplement: Supplementary file 3 — Figure S2 [file 41418_2019_443_MOESM3_ESM.jpg]

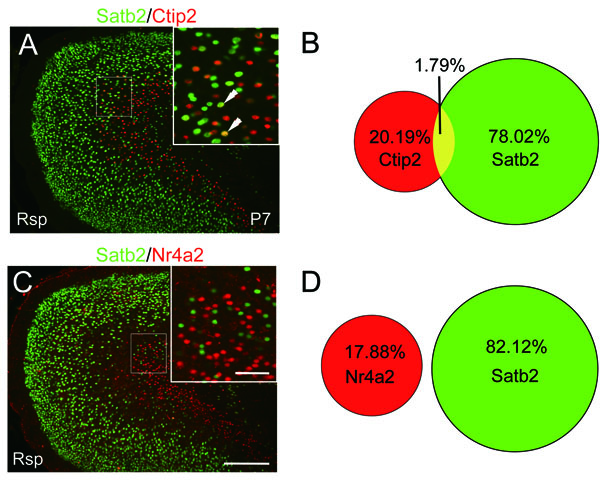

Supplement: Supplementary file 4 — Figure S3 [file 41418_2019_443_MOESM4_ESM.jpg]

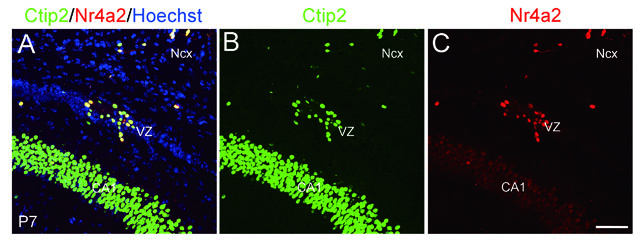

Supplement: Supplementary file 5 — Figure S4 [file 41418_2019_443_MOESM5_ESM.jpg]

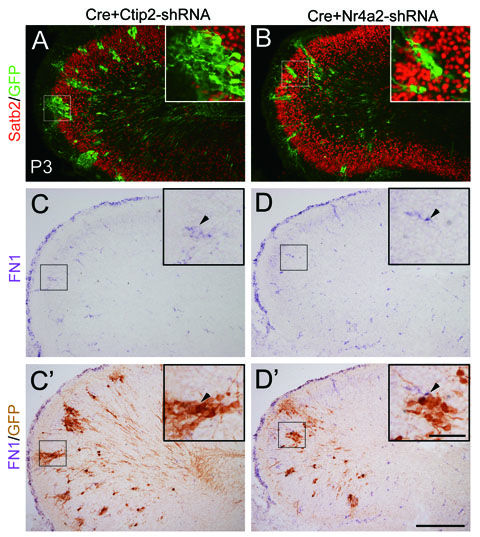

Supplement: Supplementary file 6 — Figure S5 [file 41418_2019_443_MOESM6_ESM.jpg]
